# Study of the vertically aligned in-plane switching liquid crystal mode in microscale periodic electric fields

**DOI:** 10.3762/bjnano.9.2

**Published:** 2018-01-02

**Authors:** Artur R Geivandov, Mikhail I Barnik, Irina V Kasyanova, Serguei P Palto

**Affiliations:** 1 Liquid crystals laboratory, Shubnikov Institute of Crystallography of Federal Scientific Research Centre “Crystallography and Photonics” of Russian Academy of Sciences, 119333 Leninsky pr-t 59, Moscow, Russia

**Keywords:** electrooptics, in-plane switching, liquid crystals, vertical alignment

## Abstract

The ongoing interest in fast liquid crystal (LC) modes stimulated by display technology and new applications has motivated us to study in detail the in-plane switching (IPS) vertically aligned (VA) mode. We have studied how the decrease of the period of the interdigitated electrodes (down to sub-micrometer scale) influences the switching speed, especially the LC relaxation to the initial homeotropic state. We have found that there are two types of the relaxation: a fast relaxation caused by the surface LC sub-layer deformed in the vicinity of the electrodes and the slower relaxation of the bulk LC. The speed of the fast (surface) mode is defined by half of a period of the electrode grating, while the relaxation time of the bulk depends on the LC layer thickness and the length of the driving electric pulses. Thus, the use of the surface mode and the reduction of the electrode grating period can result in significant increase of switching speed compared to the traditional LC modes, where the bulk relaxation dominates in electrooptical response. We have studied thoroughly the conditions defining the surface mode applicability. The numerical simulations are in good agreement with experimental measurements.

## Introduction

In 1997, Lee and co-workers discovered a new LC switching mode and called it vertically aligned in-plane switching (VA-IPS) mode [1]. At that time researchers were looking for LC modes with viewing angle characteristics and response times better than those of the twisted nematic mode (TN). Later a number of scientific publications featuring study of the VA-IPS mode appeared [2–4]. However, in the 2000s the agile display technology development trends turned to applications of the light-efficient fringe-field switching (FFS) mode developed by the same group of authors [5], and the VA-IPS mode was put aside. Nevertheless, the study of the VA-IPS mode in order to improve the response speed was continued. For instance, one approach is based on the addition of extra in-plane electrodes to the opposite substrate [6]. Said approach requires a precise alignment of in-plane electrodes at the opposite substrates, while the resultant benefit from the use of additional electrodes remains questionable.

In our opinion, decreasing the characteristic size of LC deformation is the key to speeding up the VA-IPS mode. Recent advances in photolithography allow for the use of sub-micrometer spatial resolution in electrode patterning. The decrease of the electrode size down to the micrometer and sub-micrometer scale opens up new possibilities for the application of the VA-IPS LC mode. When the extent of LC deformation is reduced, the switching and relaxation times are going down as well [7]. Also, a small pixel size is of special interest for holography and microdisplay applications based on LC spatial light modulators [8].

One of the questions that should be clarified regarding the in-plane interdigitated electrode parameters is related to the coupling between the deformation of the LC bulk defined by the LC layer thickness, *d*, and the surface deformation caused by the periodicity of the electrodes, *p*. Our idea is based on an assumption that if the period is smaller than the cell thickness than the penetration of high electric fields into the LC bulk will be limited by a value of *p*. Therefore, a strong and fast LC director deformation will occur only in the part of the LC layer adjacent to the electrodes, which should be favorable for a higher relaxation speed. On the other hand, the surface deformation influences the LC bulk due to elastic torques that are likely to result in elastic waves propagating into the LC volume. Involving more LC volume is not favorable for short switching times. Thus, the duration of the surface deformation existance and the length of the driving electric pulse will also become important. All these problems are new and they are addressed in detail in this work.

We show the results of our study of a new VA-IPS surface mode featuring fast switching. The experimentally obtained data are discussed together with results of numerical simulations. The experimental data and simulation results are especially important for understanding the influence of both the electrode grating geometry and the field-induced micro- and nanoscale LC deformation onto the switching dynamics.

## Results and Discussion

### Experimental LC cells study

**Figure 1:**
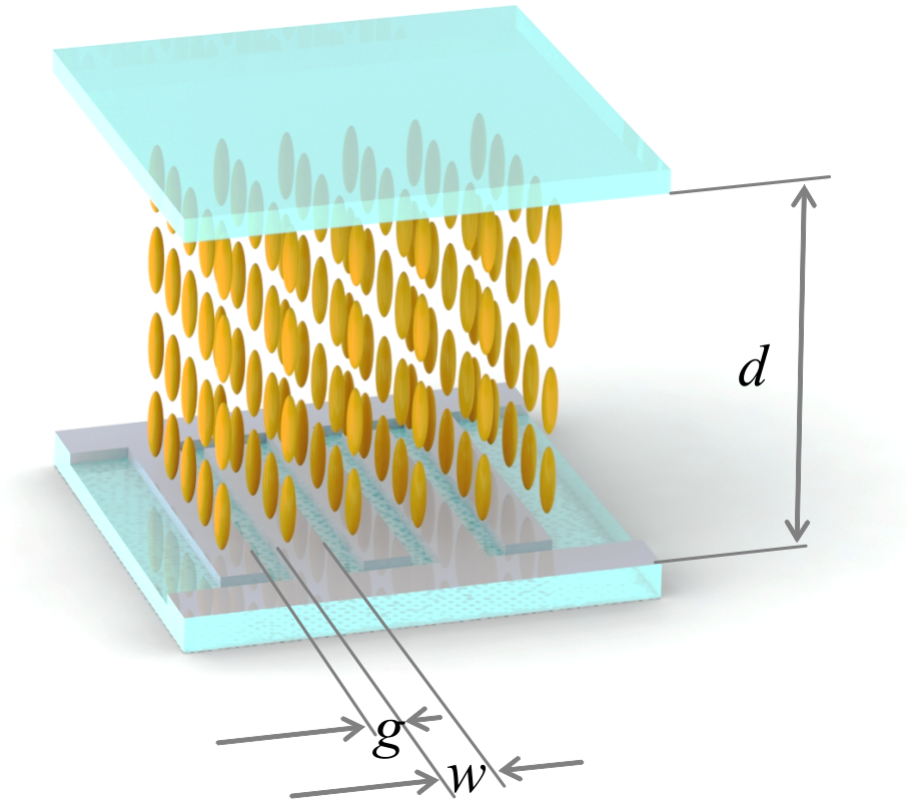
Experimental LC cell geometry.

We built an experimental LC cell as shown schematically in Figure 1. We used interdigitated electrodes with three different combinations of width, *w*, and gap between fingers, *g*: 1) *w* = 2 μm, *g* = 1 μm; 2) *w* = 2 μm, *g* = 4 μm; and 3) *w* = 4 μm, *g* = 6 μm. Thus, we have studied experimentally samples with width-to-gap ratios of *w/g* = 2.0, 0.5 and 0.66 for periods of *p* = 3, 6 and 10 μm, respectively. The electrodes were made either of transparent indium tin oxide (ITO) or opaque chromium coating prepared by vacuum sputtering.

The LC cells were filled with Merck E7 and ZLI 1957/5 LC mixtures with positive dielectric anisotropy. E7 is almost twice as viscous as ZLI 1957/5 and exhibits almost double birefringence (*∆n*). The larger birefringence of E7 allows for making smaller cells with an equivalent optical retardation. Also, the larger low-frequency dielectric anisotropy of E7 yields a stronger electric field torque, which is favorable for shortening the switching-ON time despite the higher LC viscosity.

**Figure 2:**
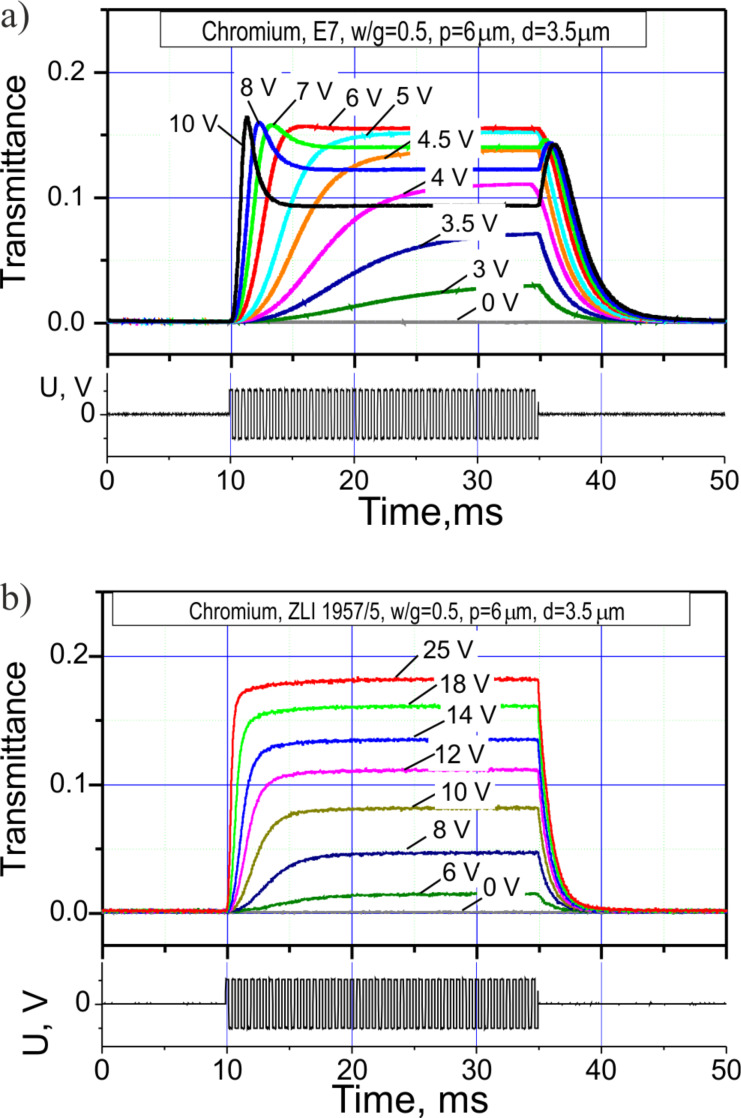
Experimentally measured electrooptical response for (a) E7 and (b) ZLI 1957/5 LC cells with chromium electrodes: *w*/*g* = 0.5 (*p* = 6 μm), *d* ≈ 3.5 μm; the driving voltage waveform is shown in the bottom graphs. The response curves show the transmittance switching for the LC cells placed between two crossed polarizers with the electrode “fingers” at 45° with respect to the absorption axes of the polarizers. The values of the transmittance are the ratio between the intensity of the transmitted beam and the intensity of the unpolarized light beam at the input.

The electrooptical response of the LC cells is shown in Figure 2. As one can see, the phase retardation of the E7 LC cell is larger than π at voltages higher than 6 V, while the ZLI 1957/5 LC cell does not exceed π even at 25 V because of the smaller *∆n*.

**Figure 3:**
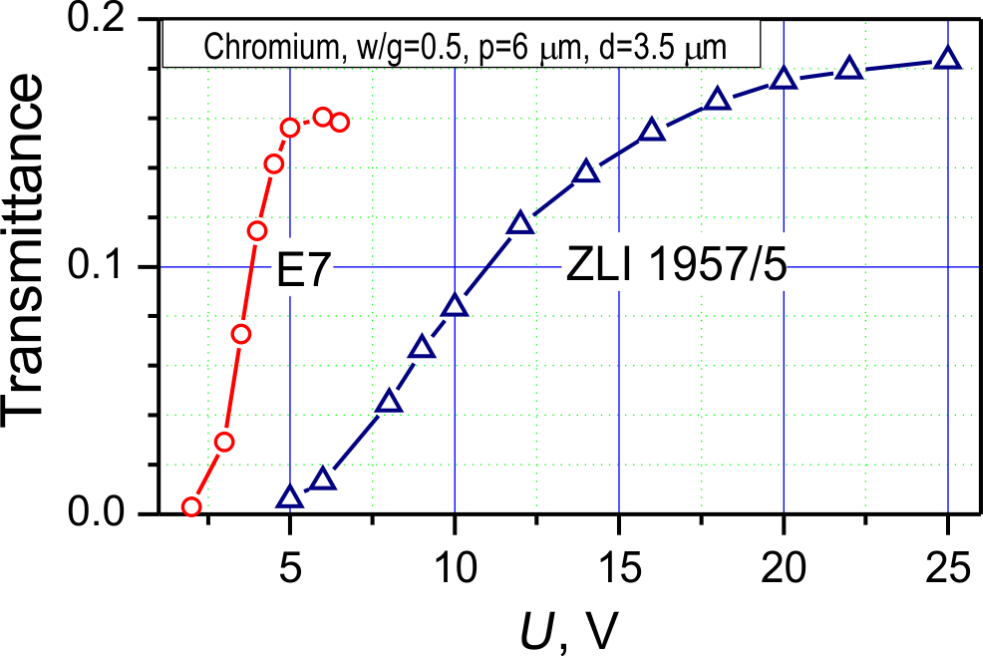
Grayscale performance of E7 and ZLI 1957/5 LC cells with chromium electrodes, *w*/*g* = 0.5 (*p* = 6 μm), *d* ≈ 3.5 μm.

From the oscillograms in Figure 2, the grayscale performance (transmittance vs voltage) plot shown in Figure 3 can be derived. Almost triple voltage is required to reach the saturation level for ZLI 1957/5 LC cell compared to E7. While the E7 LC cell is operable at driving voltage amplitudes lower than 6 V, the ZLI 1957/5 LC cell requires up to 20 V to reach the maximal transmittance.

The maximal transmittance achieved in the of ZLI 1957/5 LC cell is a few percent higher than that in the E7 cell. There are three reasons for this phenomenon: The first reason is related to the higher refractive index of the E7 LC material, which results in stronger reflections at LC–alignment layer–glass boundaries. The second reason is associated with a higher spectral dispersion of the optical phase delay, which is due to the higher birefringence of the E7 LC. The higher dispersion provides a narrower spectral band of the transmitted light in the visible range for the E7 cell. Hence, the maximal integral intensity is higher for the ZLI 1957/5 cell. Finally, there are some losses of the light energy due to light diffraction on both the electrodes and the field-induced phase grating in the LC layer. Because of the geometry of our experimental setup we measure the light intensity in a narrow angular sector (<2°) with respect to the LC layer normal. Thus, all measured results are associated with the zero-order diffraction beam. Due to the larger birefringence of the E7 LC the depth of the refractive index modulation is higher in the E7 LC cell, which can result in higher light leakage into the first and higher diffraction orders. The impact of the diffraction can also explain the higher values of transmittance we obtained by numerical simulations (see below in Figure 12) in comparison with the experimental data.

**Figure 4:**
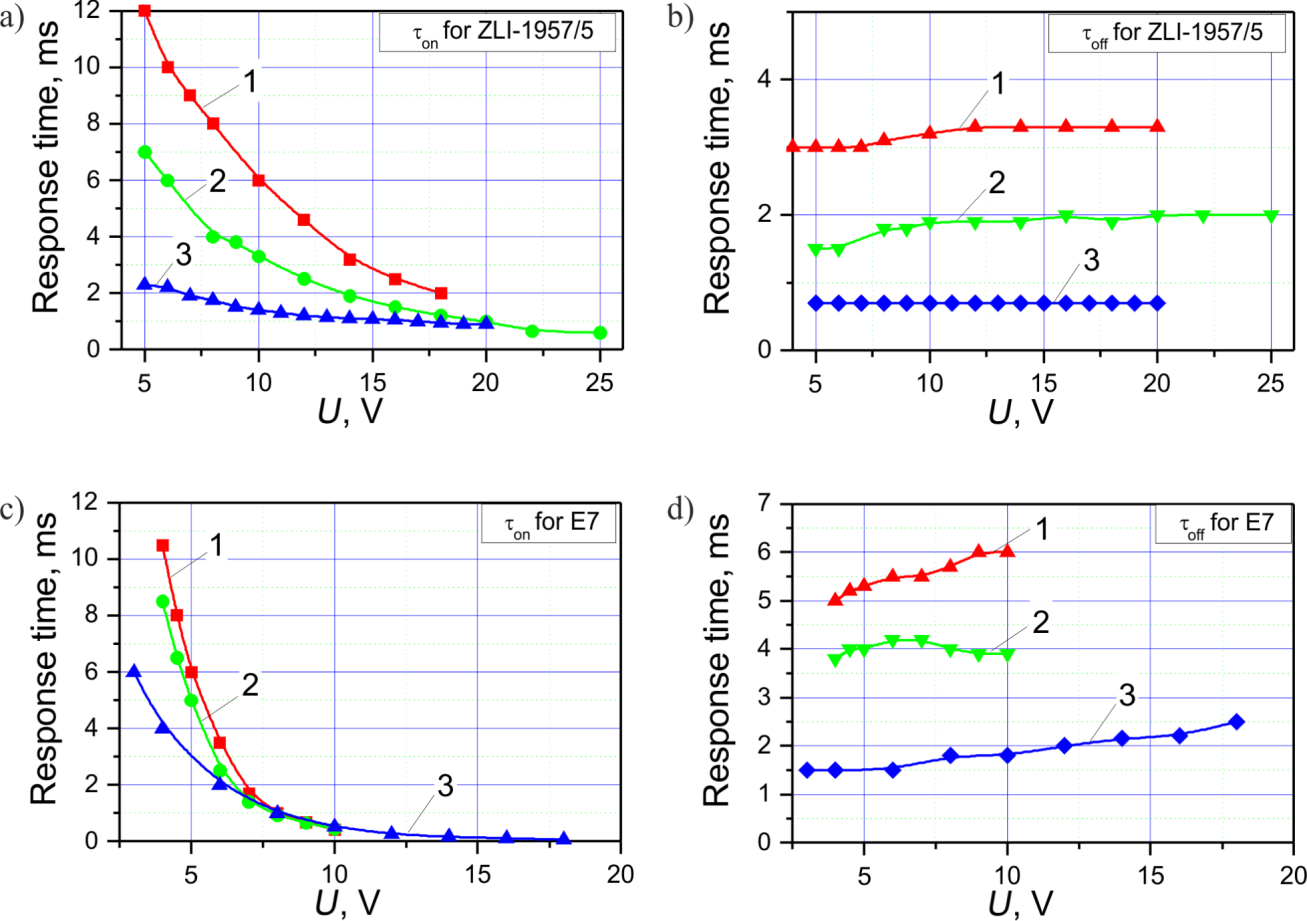
Measured response times τ_on_ (a, c) and τ_off_ (b, d) for E7 (c, d) and ZLI 1957/5 (a, b) LC mixtures in VA-IPS LC for three electrodes configurations: curve 1 – *p* = 10 μm, *w*/*g* = 0.66, *d* = 3.5 μm; curve 2 – *p* = 6 μm, *w*/*g* = 0.5, *d* = 3.5 μm; curve 3 – *p* = 3 μm, *w*/*g* = 2.0, *d* = 4 μm.

We measured the electrooptical response for three electrode periods, *p*, of 10, 6 and 3 μm for both LC materials mentioned above. Then we calculated the switching times τ_on_ and τ_off_ defined as the time interval to switch from 10% of the maximum transmittance level to 90% and vice versa. The results for the three geometries are presented in Figure 4. The increase of the electrode period causes an increase of the relaxation time. Also the switching-ON time is increased, especially at lower voltages. For all the samples the thickness of the LC layer was the same within our technological inaccuracy. Thus, the smaller electrodes period is desirable for shortening the response time. At an electrode grating period of 3 μm the relaxation time (τ_off_) is below 1 ms for ZLI-1957/5 LC. Note that for the typical modes used in practice (such as for example the twisted nematic mode) the relaxation time is defined by the thickness of the LC layer, and is significantly higher (a few tens of milliseconds for the same LC layer thickness of 3–4 μm). Of course, it is curious to know how short the relaxation can be if we further decrease the electrode gratings period to the micrometer and sub-micrometer scale. Said electrooptical performance is further discussed below in the simulation section. As one can see from Figure 4b and Figure 4d, the relaxation time tends to increase by 10% with increasing the voltage above 10 V. We relate this effect to the increased liquid crystal volume involved into the switching process.

The contrast ratio of the VA-IPS mode is limited by the contrast ratio of the polarizers and exceeds 500:1. The reasons for this high contrast ratio are the zero LC tilt angle with respect to the layer normal and the deep dark field-off state. One of the LC cell parameters important for applications is the cell transmittance. It is desirable to maximize the effective cell aperture and use optically transparent electrodes. In general, we have obtained faster switching performance in LC cells with smaller period and smaller gap size, sacrificing the maximal transmittance. As it follows from the data in Figure 3, the transmittance achieves a level of 0.18, which is quite good regarding the absorbance of the microscope polarizers (ca. 65% of the incident non-polarized light) and reflectance of the chromium electrodes. While searching a fast and light efficient LC cell design, we have also studied various geometries using our modeling software and report the results for E7 LC material in the next section.

**Figure 5:**
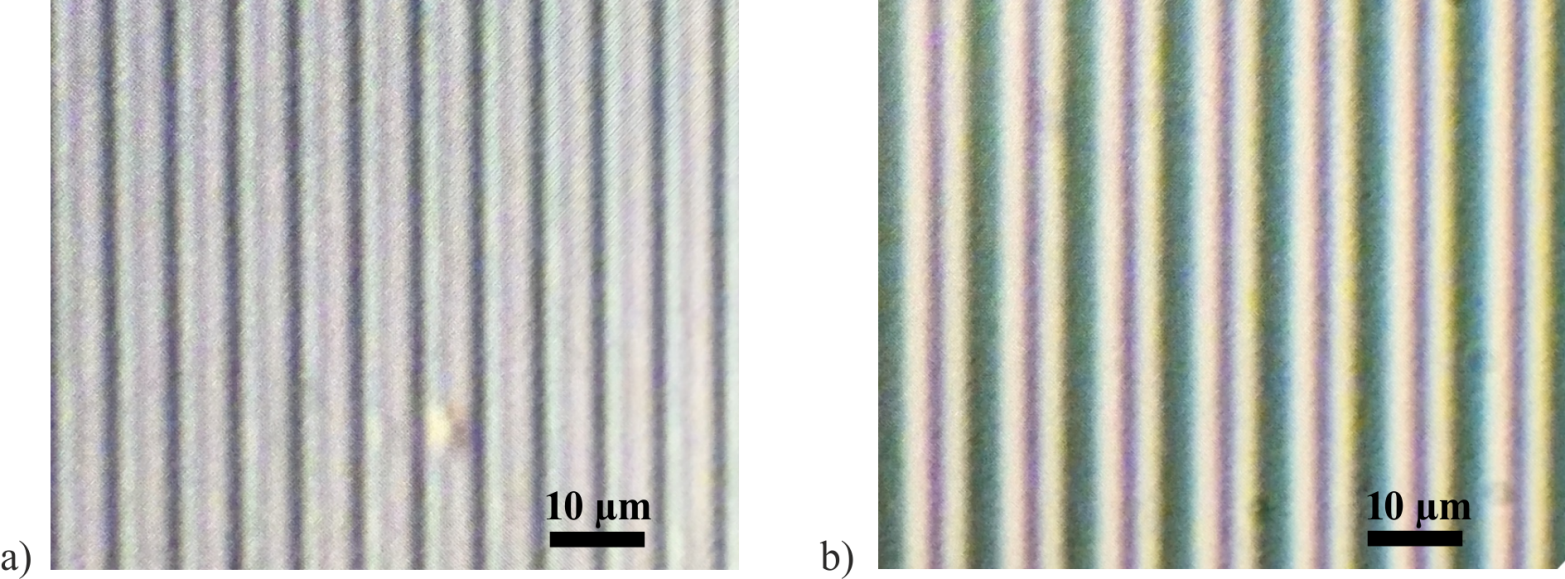
Polarized-light microscopy photographs of LC cells with electrode fingers at 45° with respect to the crossed polarizers axes at *U* = 10 V: a) *w*/*g* = 0.5, *p* = 6 μm, chromium, b) *w*/*g* = 0.66, *p* =10 μm, ITO.

In the polarized-light microscopy photos of VA-IPS cells in Figure 5 thin black stripes in the middle between the electrodes are clearly seen [9]. In Figure 5a the black stripes are quite faint compared to the black chromium electrodes, while in Figure 5b with a larger gap between the electrodes they are around 1 μm in width.

### Numerical modeling of the VA-IPS mode

**Figure 6:**
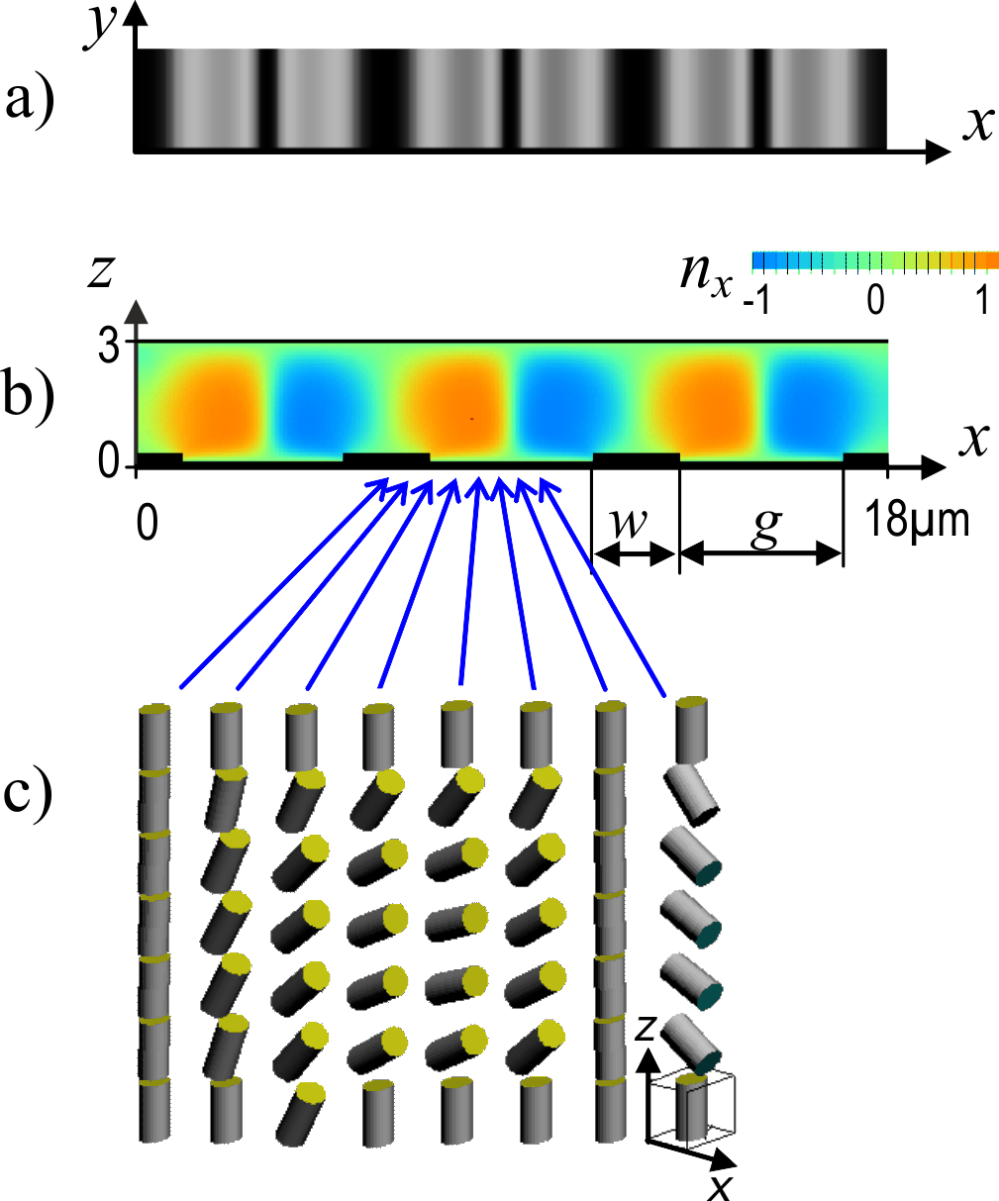
a) Top view showing the simulated local transmittance of an E7 LC cell with *d* = 3 µm and *w*/*g* = 0.5 (*p* = 6 µm), *U* = 10 V in crossed polarizers; b) *xz*-cross section of the distribution of the LC director *n_x_* (here *n_x_* = 0 corresponds to the homeotropic state); c) stick graph depicting the 2D director distribution across the cell thickness in the *xz*-plane.

The simulations are done using LCD TDK software developed by one of the authors (S. P. Palto). The software is based on solving equations of continuum LC theory for a 3D-inhomogeneous LC layer at arbitrarily defined boundary conditions. The optical calculations utilize the Berreman approach [10] with an algorithm described in [11]. The calculations are done using the E7 LC parameters listed in the “Experimental” section below. According to the simulated results for LC director distribution and local transmittance shown in Figure 6, the black stripes in the gaps between the electrodes (see Figure 5) correspond to walls with homeotropic LC alignment. Similar walls are in the middle of the electrode stripes as well. One can also compare the simulated optical image in Figure 6a with the photographs shown in Figure 5. The appearance of the homeotropic walls (also shown in Figure 6c) is related to the initial homeotropic alignment with no field and the symmetry of the electric field distribution resulting in zero electric field torque above the middle of both the electrodes and the gaps. The simulations show that in the LC volume above the electrodes the LC director field distribution is deformed only partially, which results in reduced optical phase delay and low local transmittance. A useful consequence of the homeotropic walls is that they increase the switching-off speed. The walls work as boundaries decreasing the size of the deformed volume and providing the elastic torque to make the LC director relax faster to the initial homeotropic state.

**Figure 7:**
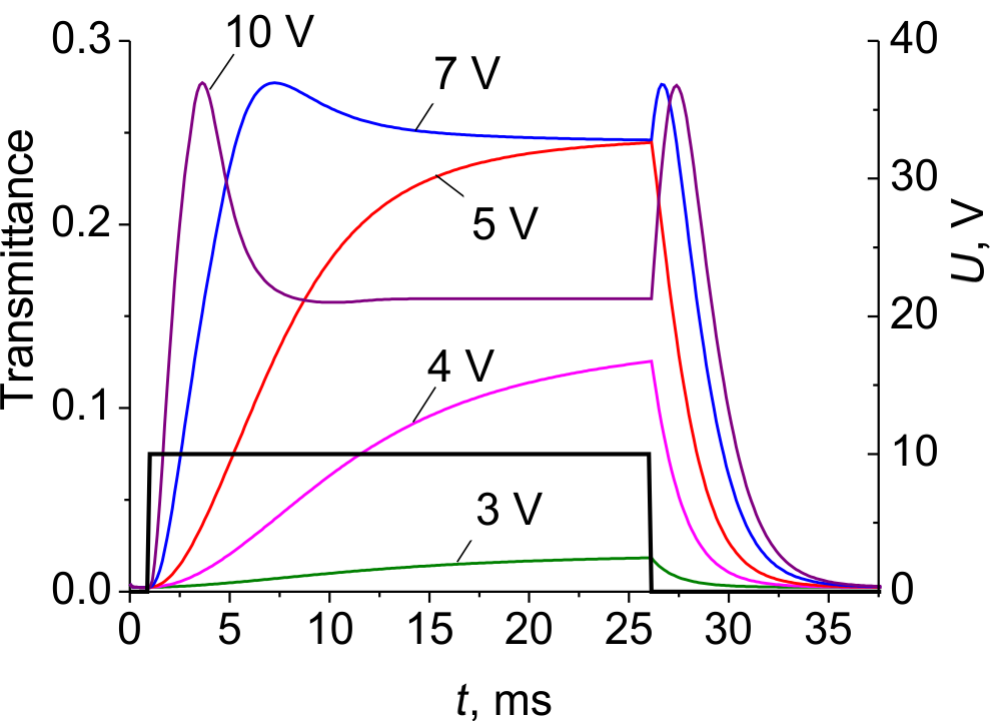
Simulated electrooptical response of an E7 LC cell with transparent electrodes: *w*/*g* = 0.5 (*p* = 6 μm), *d* = 3.5 μm to various rectangular driving voltage pulses (dark solid curve) of different amplitudes having a duration of Δ*t* = 25 ms. Here transmittance is averaged across the electrodes period *p*.

Figure 7 shows the calculated electrooptical response taking into account the inhomogeneous LC director distribution discussed above. The response results are quite similar to that observed experimentally (Figure 2a), which confirms our mathematical model.

**Figure 8:**
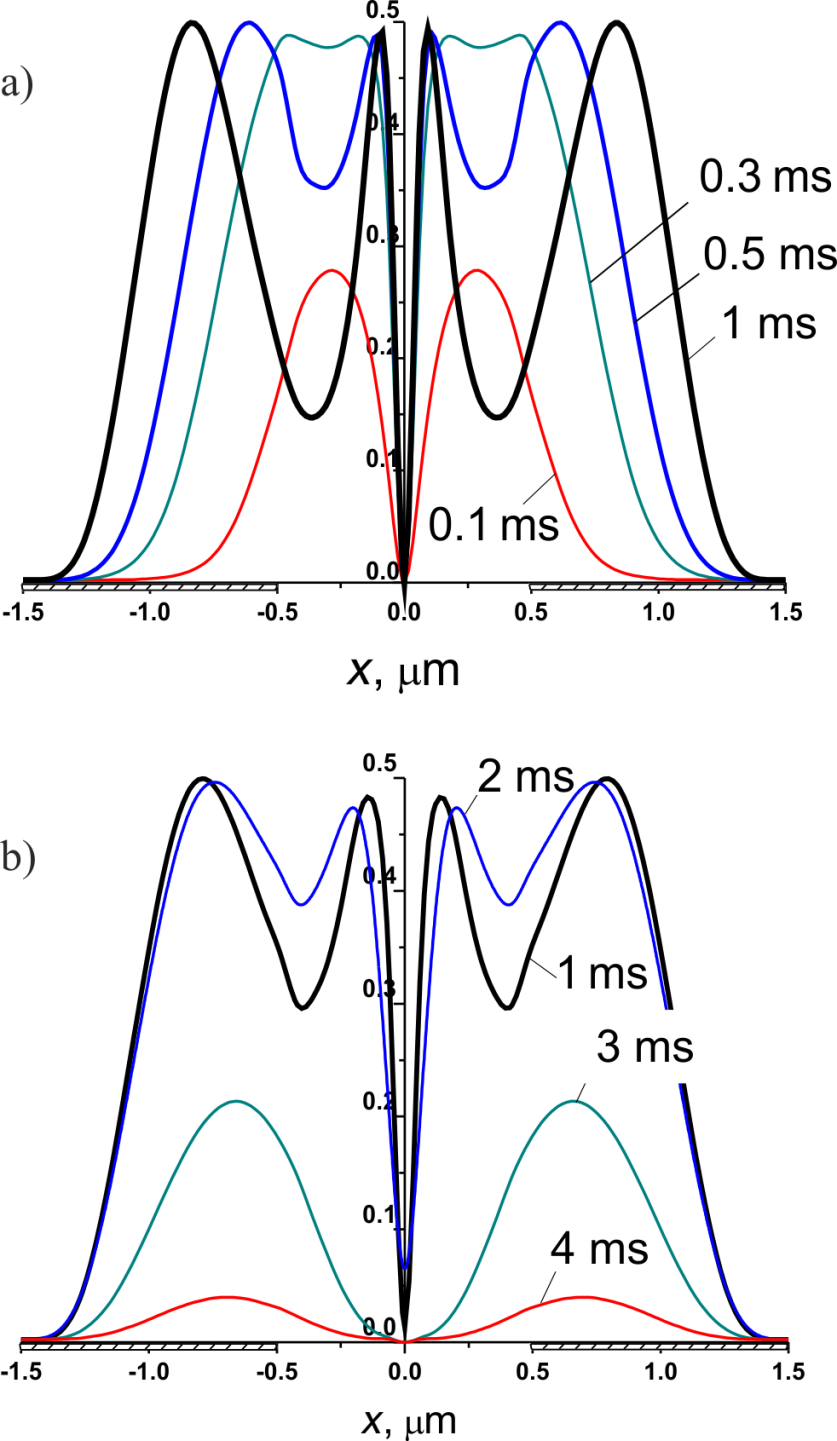
Simulated distribution of the local transmittance of an E7 LC cell with *w*/*g* = 2.0 (*p* = 3 µm) and *d* = 4 µm driven by a voltage pulse *U* = 12 V. a) 0.1, 0.3, 0.5, 1 ms after the voltage pulse is applied; b) 1, 2, 3, 4 ms after the pulse is switched off.

**Figure 9:**
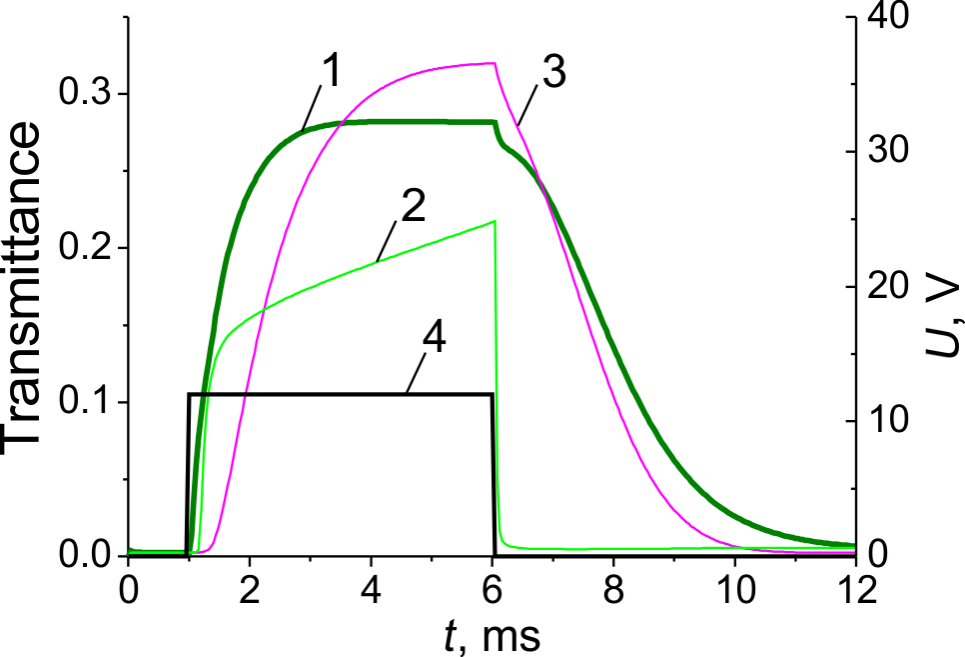
Simulated local transmittance response in an E7 LC cell with *w*/*g* = 2.0 µm (*p* = 3 µm) and *d* = 4 µm driven by 5 ms voltage pulse of 12 V. 1) Integral transmittance (local transmittance averaged over the *x*-coordinate), 2) local transmittance at point *x* = 0.1 µm, see Figure 8 (above the slit), 3) local transmittance at point *x* = 1.1 µm, see Figure 8 (above the electrode), 4) applied voltage pulse *U* = 12 V, Δ*t* = 5 ms.

In Figure 8, a series of local transmittance distribution curves of an E7 LC cell is shown at different times after the voltage pulse (*U* = 12 V, Δ*t* = 25 ms) is applied. The response is symmetric with respect to the middle homeotropic wall between the electrodes at *x* = 0. The local transmittance is distributed inhomogeneously along the *x*-axis. An optimal intensity distribution occurs, when the integral intensity is close to the maximum, which appears at times *t* of about 0.3–0.5 ms after the voltage is switched on. Later the optical phase delay exceeds π, and the transmittance is decreased above the slit. From Figure 9 one can see that above the slit the on/off switching process is much faster than above the electrodes.

**Figure 10:**
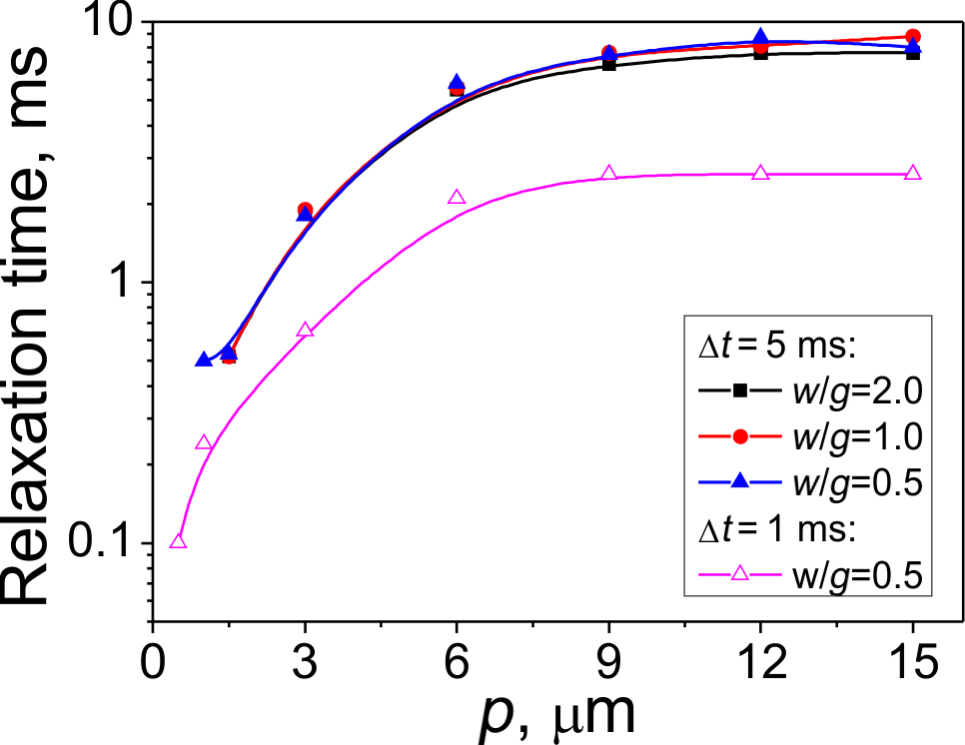
Relaxation time of an E7 LC layer of thickness *d* = 3.5 µm as a function of *p* for different ratios *w*/*g* and pulse lengths, Δ*t*, at pulse amplitude *U* = 10 V.

Figure 10 shows the relaxation (switching-off) time for different width-to-gap ratios (*w*/*g* = 2.0, 1.0 and 0.5) as a function of the electrode gratings period, *p*. The calculated data are in agreement with the experimental results showing that smaller values of *p* result in faster relaxation. The dependence on *p* is rather complicated. In a range of *p* from 3 to 6 µm (which is comparable to the LC layer thickness) the relaxation time decreases linearly as we decrease the electrode grating period. Only when the electrodes period is higher than the LC layer thickness the switching-off time tends to saturate. At short gratings periods the switching is very fast (it is well below 1 ms if *p* <1.5 µm), and there is a quadratic dependence of the switching time on *p*. It is also intriguing that at a fixed value of *p* the dependence of the switching time on the gap is rather weak.

To understand what is going on, it is useful to compare the switching-off time with that characteristic for a LC layer of the same thickness, but driven by a normal electric field created between two solid electrodes on opposite sides of the LC layer. In the last case the LC director field remains homogeneous in the *xy*-plane, so the relaxation time can approximately be estimated with the well known formula:

[1]
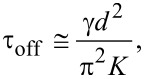


where γ is the rotational viscosity and *K* is an effective elastic constant. The estimation from Equation 1 for a viscosity of E7, *K* ≈ 10 pN and *d* = 3.5 μm yields a value of about 25 ms. If we compare this relaxation time value with the results in Figure 10 than we find that it is still significantly higher than the time for the samples of large gratings periods comparable with the layer thickness. Thus, the elastic deformation along the *x*-direction plays a very important role. This deformation can be treated in terms of a superposition of different elastic waves characterized by different spatial frequencies. While the elastic deformation along the *z*-direction (normal to the LC layer) is characterized by a spatial period equal to the double thickness (2*d*) of the layer, the spatial frequencies of the elastic waves along the *x*-direction are defined by *p*. The relaxation times for these waves related to different spatial harmonics are:

[2]
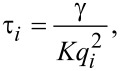


where *q_i_* = 2π*i*/*p* (with *i* being a natural number) are the spatial frequencies of the harmonics associated with the principal frequency at *i* = *1* (*q_1_* = 2π/*p*) of the electrode grating. However, due to appearance of the homeotropic walls discussed above, the most significant contribution to the deformation along the *x*- axis comes from the second harmonic (*i* = 2). This is why even at an electrode period of 7 μm the electrooptical switching-off time is still significantly lower than the time estimated for *d* = 3.5 μm from Equation 1.

**Figure 11:**
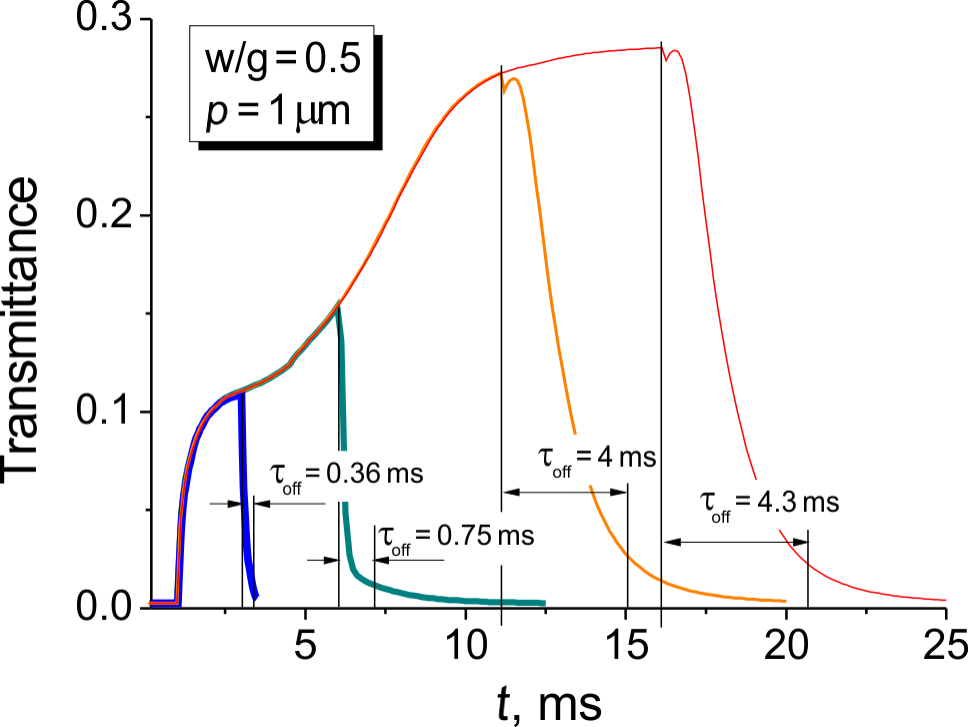
Simulated electrooptical response for rectangular pulses of duration Δ*t* = 2, 5, 10 and 15 ms applied starting from time moment *t*_pulse_ = 1 ms. Sample: E7 LC cell with *p* = 1 µm, *w*/*g* = 0.5, *d* = 3.5 µm, *U* = 10 V.

The calculations in Figure 10 correspond to two fixed driving pulse lengths of Δ*t* = 1 ms and Δ*t* = 5 ms. The length of the driving pulse becomes very important in the case of the complex 2D deformation we deal with. Decreasing the pulse length, Δ*t*, results in a significant decrease of the switching-off time. At a length of 1 ms and with the sub-micrometer electrode period, a switching-off time below 0.1 ms can be achieved. The influence of the driving pulse length is very well illustrated by the data for *p* = 1 μm in Figure 11. If the pulse duration is 2 ms then the switching-off is very fast (ca. 0.4 ms), because it is basically defined by the relaxation time of near-surface *x*-deformation with the spatial frequency of the second harmonic (*q*_2_ = 4π/*p* ≈ 12 μm^−1^). Increasing the pulse length results in a significant increase of the relaxation time (ca. 4 ms at a pulse length of 15 ms), which is the consequence of propagation of the surface deformation along the *z*-axis into the bulk. As it was discussed above, this bulk deformation has a maximum relaxation time dependent on the layer thickness, which, in our particular case, is rather high (3.5 μm). The appearance of the bulk deformation results in higher transmittance.

In Figure 11, one can also see that at the particular driving voltage, the time necessary to establish the equilibrium 2D deformation providing a transmittance level of 0.28 is about 10 ms, while the surface *x*-deformation (transmittance level ca. 0.1) is established 1 ms after the voltage is applied.

Thus, using the short driving pulses one can utilize the surface mode, which can provide very fast electrooptical switching. However, we have to pay for the speed by lower transmittance, especially if the LC material has low optical anisotropy. In the mentioned example of the 2 ms driving pulse the maximal switched-on transmittance is about 10%, which is a fifth of the 50% in the ideal case (at 100% optical aperture, ideal polarizers and π-phase delay provided by LC layer; we are also reminding that we define the transmittance with respect to the intensity of the unpolarized beam at the input), but it is still sufficiently high for many applications.

**Figure 12:**
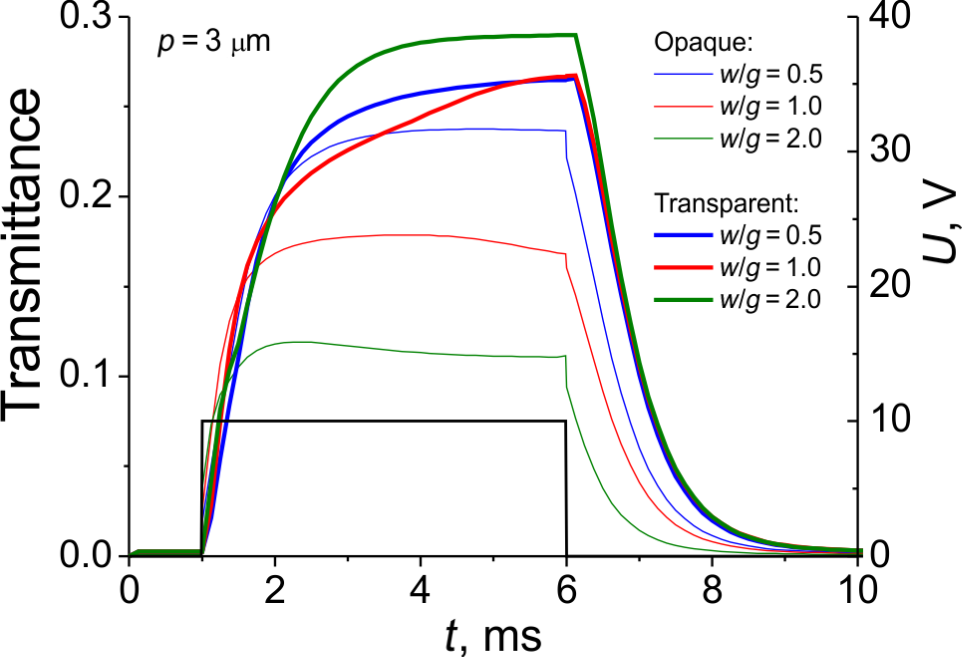
Simulated electrooptical response for E7 LC cells with transparent and opaque electrodes (*p* = 3 µm and *d* = 3.5 µm) at *U* = 10 V, rectangular pulse length Δ*t* = 5 ms, *w*/*g* = 0.5, 1.0 and 2.0.

Finally, we would like to discuss the optimal geometry and the influence of the optical transparency of the electrodes grating on the electrooptical performance. As an example, let us consider the case of an E7 LC cell with period *p* = 3 µm and a pulse duration of 5 ms. From Figure 12 we can see that for opaque electrodes the highest average transmission is reached at *w*/*g* = 0.5. In contrast, for transparent electrodes the best transmittance is achieved for the highest ratio *w*/*g* = 2.0. It is important to note, that all the curves of the “transparent” series lie above those of the “opaque” series, apparently due to some additional deformation of the LC director above the electrodes (see Figure 8 for details). Given this, along with the fact that the relaxation time is almost similar for all the curves, we can conclude that the use of the transparent electrodes is preferable.

## Conclusion

We have studied the electrooptical performance of vertically aligned LC as a function of the in-plane electrodes geometry. Reducing the electrode period allows for sub-millisecond response times, becoming as short as 100 µs at a period of 500 nm. The results are discussed in terms of different relaxation modes that define the speed of the electrooptical switching. The so-called surface mode is due to the periodic structure of the surface electrode system. The spatially periodic electric field near the surface results in appearance of the homeotropic walls in LC director distribution. These walls double the spatial frequency of the LC elastic deformation and in the case of small spatial periods provide very fast LC director relaxation. Another relaxation mode is caused by propagation of the elastic deformation from the surface into the bulk. The relaxation time of the bulk mode depends on the penetration depth of the deformation into the LC layer volume along the layer normal. This depth can be controlled by both the driving pulse duration and the thickness of the LC layer. By varying the electrodes width-to-gap ratio we have found the LC cell parameters enabling both high transmittance and fast response times. The VA-IPS mode with transparent microelectrodes is a perspective for display application requiring small pixel size and fast response time.

## Experimental

Samples preparation: We used interdigitated electrodes with three sets of width, *w*, and gap between fingers, *g*: 1) *w* = 2 μm, *g* = 1 μm; 2) *w* = 2 μm, *g* = 4 μm; and 3) *w* = 4 μm, *g* = 6 μm. Thus, we studied samples with width-to-gap ratios *w*/*g* = 2.0, 0.5 and 0.66 for periods *p* =3, 6 and 10 μm, correspondingly. The electrodes were made either of transparent indium tin oxide (ITO) or opaque chromium coating prepared by vacuum sputtering. Using the electrodes substrate and another glass substrate without electrode we assembled a number of LC cells with varying thickness *d* from 2 to 4 μm. The LC alignment was made using a chromolane alignment layer spin-coated onto the inner substrates surfaces and baked at 90 °C for 30 min. The LC cells were filled at room temperature using the following LC mixtures: 1) Merck E7 LC material (*n*_e_ = 1.7462, Δ*n* = 0.2246 at λ = 589 nm, 20 °C; ε_||_ = 19.0 (at *f* = 1 kHz), Δε = +13.8, γ = 0.19 Pa·s at 20 °C, *K*_1_ = 11.1 pN, *K*_2_ = 9.0 pN, *K*_3_ = 17.1 pN) [12], and 2) Merck ZLI1957/5 LC material (*n*_e_ = 1.6240, Δ*n* = 0.1213 at λ = 589 nm, 20 °C; ε_||_ = 8.0 (at *f* = 1 kHz), Δε = +4.5, γ = 0.105 Pa·s at 20 °C, *K*_1_ =14 pN, *K*_2_ = 7 pN, *K*_3_ = 16 pN) [13].

Electrooptical measurements were performed under a polarizing microscope Olympus CX31PF. The light spectrum from the halogen microscope lamp was narrowed using a glass filter with a transmission range of 320–680 nm.

The driving voltage from arbitrary waveform generator connected in series with custom-built amplifier based on Apex Microtechnology PA94 chip featuring high voltage ±450 V and high slew rate 500 V/µs was applied to the sample electrodes. The rectangular waveform was of an amplitude varied from 0 to 25 V at the frequency *f* = 2 kHz. An LC cell was placed at the microscope stage between the crossed analyzer *A* and input polarizer *P*. The cell was rotated to the angular position in which the electrode grating wavevector coinciding with the projection of the electric field, *E*, is oriented at 45° to the polarizers extinction axes. The electrooptical response was recorded using a photomultiplier connected to a digital oscilloscope. The optical images in polarized light were taken using Olympus OM-D EM5 camera attached to the microscope C-mount using an adapter.

The numerical simulations of three-dimensional LC director distribution above patterned electrodes and corresponding optical performance of LC cell were calculated using LCD TDK 3.0 software developed by S. P. Palto. The LCD TDK 3.0 is based on the Ericksen–Leslie theory and the Berreman 4 × 4 matrix approach for solving the optical problem. The calculations of the electrooptical response are carried out for the central wavelength of the visible spectrum (λ = 550 nm). The real LC parameters mentioned above were used for modeling.
